# *E. coli* HflX interacts with 50S ribosomal subunits in presence of nucleotides

**DOI:** 10.1016/j.bbrc.2008.12.072

**Published:** 2009-02-06

**Authors:** Nikhil Jain, Neha Dhimole, Abu Rafay Khan, Debojyoti De, Sushil Kumar Tomar, Mathew Sajish, Dipak Dutta, Pradeep Parrack, Balaji Prakash

**Affiliations:** a Department of Biological Sciences and Bioengineering, Indian Institute of Technology, Kanpur 208016, India; b Department of Biochemistry, Bose Institute P-1/12, C.I.T. Scheme VIIM, Kolkata 700054, India

**Keywords:** HflX, GTPase, ATPase, rRNA binding protein, Ribosome binding GTPase, Ribosome assembly

## Abstract

HflX is a GTP binding protein of unknown function. Based on the presence of the *hfl*X gene in *hfl*A operon, HflX was believed to be involved in the lytic-lysogenic decision during phage infection in *Escherichia coli*. We find that *E. coli* HflX binds 16S and 23S rRNA – the RNA components of 30S and 50S ribosomal subunits. Here, using purified ribosomal subunits, we show that HflX specifically interacts with the 50S. This finding is in line with the homology of HflX to GTPases involved in ribosome biogenesis. However, HflX-50S interaction is not limited to a specific nucleotide-bound state of the protein, and the presence of any of the nucleotides GTP/GDP/ATP/ADP is sufficient. In this respect, HflX is different from other GTPases. While *E. coli* HflX binds and hydrolyses both ATP and GTP, only the GTP hydrolysis activity is stimulated by 50S binding. This work uncovers interesting attributes of HflX in ribosome binding.

## Introduction

GTP binding proteins are well known regulators of diverse cellular processes. Several of these proteins are well characterized, and are termed molecular switches. They switch from the ‘empty’ nucleotide free state to an ‘ON’ state upon GTP binding and a subsequent GTP hydrolysis leads to the GDP bound ‘OFF’ state [1,2]. Although a similar switching mechanism prevails in a set of 11 universally conserved bacterial GTPases [3], an understanding of their biological functions is only beginning to emerge.

Gene products of the *hfl*A operon, including HflX, are thought to play a role in lytic-lysogeny decision of coliphage lambda during phage infection in *Escherichia coli* [4]. In a recent work, we negate such a role for HflX, despite its presence in this operon (to be published elsewhere). However, the widespread conservation of the *hflX* gene across species led us to investigate alternative roles for HflX based on the high sequence homology it shares with the members of ODN (Obg, DRG1 and Nog1) family, which have been implicated in ribosome assembly [5].

HflX is one of the 11 conserved bacterial GTPases. Several of them are reported to bind ribosomal subunits, largely in a nucleotide specific manner and take part in ribosome biogenesis or assembly [3,9–14]. In their GTP bound states, the circularly permuted GTPases [6] YqeH, YloQ and YjeQ in *Bacillus subtilis* bind the 30S subunit (Anand et al., unpublished results) [7,8] while YlqF binds the 50S. YlqF and YqeH have been implicated in ribosome assembly [9,10]*.* YphC (EngA homologue in *B. subtilis*) and YsxC bind 50S in a GTP-dependent manner and together function in 50S assembly [11,12]. The only exception is Era, which binds the 30S subunit in the nucleotide free state [13]. Also, *E. coli* Obg is the only known protein that interacts with both 30S and 50S subunits [14]. Nog1 and DRG1 are eukaryotic GTPases that participate in 60S assembly [15,16]. However, the biochemical characterization of HflX that shares a high homology with Obg and Nog1 has not been reported until recently.

In this work, we show that like Obg, DRG1 and Nog1, HflX too interacts with the large ribosomal (50S) subunit. While this manuscript was under preparation, an association between HflX from *Chlamydophila pneumoniae* (cpHflX) and 50S subunits from *E. coli* has been reported [17]. In the present report, however, we demonstrate an interaction between *E. coli* HflX and purified *E. coli* ribosomal subunits. Additionally, we find that this interaction can take place in the presence of GTP as well as in the presence of other nucleotides such as GDP, ATP or ADP. This is in contrast to most ribosome binding GTPases that preferentially associate with 50S/30S only in their GTP bound forms. Furthermore, we find that full length HflX is required for HflX-50S interaction – an attribute akin to most ribosome binding GTPases. Interestingly, HflX is not only a GTPase, but it also binds and hydrolyses ATP efficiently (to be published elsewhere). However, 50S binding stimulates only the GTP hydrolysis (albeit moderately), but not ATP hydrolysis.

## Materials and methods

*Cloning, expression and purification of HflX.* Recombinant *E. coli* HflX and deletion constructs ΔN-HflX, ΔC-HflX and HflX-G proteins were cloned, expressed and purified to homogeneity (for details, see supplementary material). Purified proteins were concentrated, aliquoted and stored at −80 °C after snap freezing in liquid nitrogen, for further use.

*Gel retardation assays.* These assays were performed by incubating HflX with 16S and 23S rRNA at 37 °C for 15 min in the presence or absence of GMPPNP/GDP (Sigma–Aldrich). Twenty percent Glycerol was added to the reaction mixture and was analyzed by native agarose gel stained with ethidium bromide.

*Ribosome purification.* BL21 cells, grown till 0.6 OD_600_ at 37 °C and incubated on ice for 10 min after addition of 100 μg/mL chloramphenicol, were harvested by centrifugation and were lysed by 5 cycles of freeze-thaw in Buffer D (20 mM Tris–HCl pH 8.0, 50 mM NH_4_Cl, 5 mM MgCl_2_, 1 mM DTT, 1 mg/mL lysozyme, protease inhibitor cocktail). Following the addition of RNase free DNase, the lysate was clarified by centrifugation at 45,000*g* at 4 °C. Supernatant was loaded on a 1.1 M sucrose cushion and centrifuged at 50,000 RPM for 4 h at 4 °C (Sorvall-TH660 rotor). The pellet was dissolved in Buffer E (20 mM Tris–HCl pH 8.0, 50 mM NH_4_Cl, 1 mM Mg-Acetate, 1 mM DTT). The ribosome thus obtained was stored at −80 °C.

Ribosomal subunits were purified by loading the supernatant (as mentioned above) on 18–50% sucrose step gradient and centrifuged at 28,000 RPM (Sorvall Surespin-630 rotor) at 4 °C for 10 h. Gradient was fractionated by upward displacement using 60% sucrose by ISCO density gradient fractionator. RNA isolated from each fraction was analyzed by formaldehyde agarose gel (1.5% agarose)*.* Fractions containing 30S and 50S subunits were identified based on the presence of 16S and 23S rRNA, respectively. Fractions corresponding to these subunits were pooled separately and diluted by the addition of Buffer F (20 mM Tris–HCl pH 8.0, 50 mM NH_4_Cl, 10 mM Magnesium Chloride, 1 mM DTT). Ribosomal subunits were then concentrated using Millipore Amicon ultra centrifugal filter tubes.

While using them in ATP/GTP hydrolysis assays, 50S was diluted in Buffer G (20 mM Tris–HCl pH 8.0, 200 mM NH_4_Cl, 1 mM Mg-Acetate, 1 mM DTT) and precipitated by centrifuging at 45,000 RPM at 4 °C for 4 h (Sorvall-TH660 rotor). Pellet, thus obtained, was washed with 1 M NH_4_Cl and dissolved in a small amount of Buffer G, and stored at −80 °C.

*Protein–ribosome co-sedimentation analysis.* Purified protein and ribosomes were incubated at 37 °C for 30 min in Buffer E in presence of 2 mM nucleotides (GDP/GMPPMP) and loaded on a 20–43% Sucrose gradient in Buffer F. The tubes were centrifuged at 28,000 RPM for 10 h (Sorvall surespin-630 rotor) and 400 μl fractions collected from the top of the tube were analyzed by SDS–PAGE. Protein was detected by immuno-blotting using anti-His antibody (Santacruz). As above, the presence of 50S/30S in each of the fractions was determined by the presence of 16S rRNA (∼1.5 Kb band) and 23S rRNA (∼2.9 Kb band). Co-sedimentation experiments with purified 50S and 30S subunits were carried out similarly, except replacing the sucrose gradient to 18–50%.

*GTP and ATP hydrolysis assays.* GTP hydrolysis assays were carried out in 5 μl reaction volumes containing 20 μM HflX, 50 mM Tris–HCl pH 8.0, 200 mM NaCl, 1 mM DTT, 5 mM MgCl_2_, 20 μM GTP, 1 μCi γ[^32^P] GTP, and were incubated at 37 °C for 60 min. The reaction was stopped by adding 1 μl of 6 M formic acid and centrifuged at 13,000 RPM for 10 min. Five microliters of the sample was spotted on the PEI-coated TLC (Merck), resolved in 1.5 M KH_2_PO_4_ (pH 3.4) buffer and subjected to autoradiography to detect the formation of inorganic PO_4_. Autoradiograms were aligned with the TLC plate and spots corresponding to inorganic PO_4_ were cored out. The counts (CPM) were determined using a scintillation counter. For ribosome stimulation assays, varying amounts of 50S (0–20 pmoles) were used along with. In the competition assays, GTP hydrolysis (without the ribosome) was carried out using 0–500 μM ATP/AMPPNP/ADP. Similarly, for ATP hydrolysis assays GTP was replaced by ATP and competed with 0–500 μM GTP/GMPPNP/GDP. In competition assays, CPM obtained in absence of competitor was normalized to 100%. Percent activities for the other samples were calculated with respect to this.

## Results

### HflX binds ribosomal RNA

HflX shares a high sequence homology with Obg, DRG1 and Nog1 that belong to the family of ribosome binding GTPases, some of which also interact with the ribosomal RNA. This led us to investigate if HflX too binds rRNA and/or the ribosome [11,13,14,19].

**Fig. 1:**
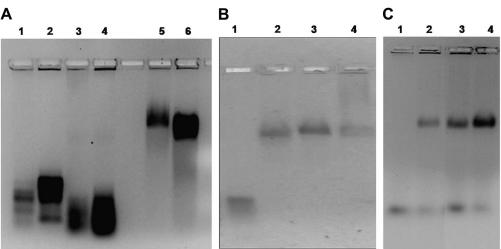
HflX interacts with 16S and 23S rRNA in a nucleotide independent manner. HflX was incubated with 16S and 23S rRNA in the presence or absence of GMPPNP/GDP and was analyzed on 1% native agarose gel. (A) Interaction of rRNA with HflX and BSA (negative control). Lane 1, 16S rRNA; Lane 2, 23S rRNA; Lane 3, 16S rRNA + BSA; Lane 4, 23S rRNA + BSA; Lane 5, 16S rRNA + HflX; Lane 6, 23S rRNA + HflX. (B) HflX-23S rRNA interaction in presence of nucleotides. Lane 1, 23S rRNA; Lane 2, 23S rRNA + HflX; Lane 3, 23S rRNA + HflX + GDP; Lane 4, 23S rRNA + HflX + GMPPNP. (C) HflX-16S rRNA interaction in presence of nucleotides. Lane 1, 16S rRNA; Lane 2, 16S rRNA + HflX; Lane 3, 16S rRNA + HflX + GDP; Lane 4, 16S rRNA + HflX + GMPPNP.

The *hfl*X gene from *E. coli* was cloned into pET28a vector, and the His_6_-tagged fusion protein was overexpressed and purified to near homogeneity (see Materials and methods). Gel retardation assays show a clear shift in the mobility of 16S and 23S rRNA in presence of HflX (Fig. 1A), in a nucleotide independent manner (Fig. 1B, C). A similar effect was not observed with BSA that served as a negative control (Fig. 1A, lanes 3 and 4). In contrast, rRNA binding to Obg is nucleotide specific [14].

### GTP hydrolysis by HflX is inhibited in presence of ATP

**Fig. 2:**
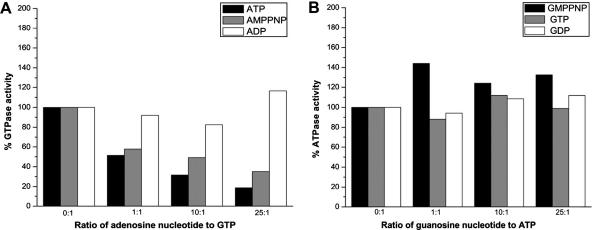
GTP hydrolysis by HflX is inhibited in presence of ATP. Hydrolysis of radiolabelled [γ^32^P]-ATP, [γ^32^P]-GTP was studied in presence of 0- to 25-fold excess of unlabelled nucleotides in a competition experiment. Five microliters reaction mixture was spotted on PEI-TLC plates, separated and visualized as described in ‘Materials and methods’. Ratio of competing nucleotide to ATP/GTP is indicated on *x*-axis. (A) GTP hydrolysis in presence of ATP, AMPPNP and ADP. Amount of GTP hydrolysed, in the absence of competing nucleotides was normalized to 100%. (B) ATP hydrolysis in presence of GMPPNP, GTP and GDP. Amount of ATP hydrolysed, in the absence of competing nucleotides was normalized to 100%.

HflX displays a characteristic G domain with G1–G4 motifs required for binding and hydrolyzing GTP (Fig S1, Supplementary material). Nevertheless, *E. coli* HflX also binds and hydrolyses ATP. Interestingly, the rate of ATP hydrolysis is higher than that of GTP hydrolysis (to be published elsewhere). As HflX contains a single nucleotide binding domain, i.e. the G-domain, we wished to examine if ATP too binds the same site. As shown in Fig. 2A, the rate of GTP hydrolysis reduced to ∼50% when either ATP or AMPPNP (a non-hydrolysable ATP analog) was present at concentrations equal to that of GTP, and to ∼20% when the latter were in 25-fold molar excess. Interestingly, a similar effect was not observed in presence of ADP (Fig. 2A). The ATP hydrolysis rates, however, remained unaffected in the presence of GTP, GMPPNP or GDP (Fig. 2B).

### Full length HflX binds the 50S ribosomal subunit regardless of the bound nucleotide

**Fig. 3:**
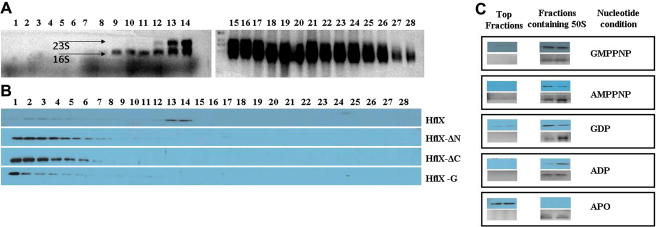
HflX associates with the 50S subunit. HflX-ribosome co-fractionation experiments were conducted by incubating purified HflX and ribosomes from *E.coli*, in presence of GMPPNP, a non-hydrolysable GTP analog. This mixture was loaded on a 20–43% sucrose gradient, centrifuged and fractionated as described in Materials and methods. Each fraction was analyzed for the presence of RNA and protein. (A) A formaldehyde agarose gel of the RNA isolated from each of the fractions depicts the presence of 23S and 16S rRNA, as indicated. (B) His_6_-tagged HflX or the truncated proteins present in these fractions were detected by western blotting using an anti-His antibody. HflX refers to the full length protein, and ΔN-HflX (193–426), ΔC-HflX (1–362) and HflX-G (193–362) correspondingly refer to the fragments lacking the N and C terminal domains or both. (C) Co-fractionation experiments were repeated with purified HflX and the 50S subunit in presence of various nucleotides (shown in separate panels and indicated on the right). Unlike in B, the top fractions devoid of 50S and only the peak fractions containing 50S are shown (top gel, in blue color), based on the presence of 23S rRNA in these fractions (lower gel in grayscale). HflX co-fractionates with 50S in presence of GMPPNP, AMPPNP, GDP and ADP, but not in absence of nucleotides (Apo). (For interpretation of the references to color in this figure legend, the author is referred to the web version of the article.)

As HflX binds both 16S and 23S rRNA (Fig. 1), we set out to examine its association with 30S as well as 50S subunits. At the outset, ribosome co-sedimentation studies were carried out employing crude ribosomes (consisting of 50S, 30S, 70S and polysomes) and HflX in presence of GMPPNP, a non-hydrolysable GTP analog. This was layered on 18–43% sucrose gradient and fractions were collected following ultracentrifugation (see Materials and methods). The presence of various ribosomal subunits in these fractions was assessed based on the rRNA content (Fig. 3A). HflX was detected in fractions where the 50S subunit begins to appear (Fig. 3B).

Sequence analysis (Fig. S1, Supplementary material) revealed the presence of three domains in *E .coli* HflX. In order to assess the role of the various domains of HflX in ribosome binding, we created HflX constructs lacking the N-terminal domain (ΔN-HflX), the C-terminal domain (ΔC-HflX) or both (HflX-G). Co-sedimentation experiments show that in all the three truncated proteins, ribosomal interactions were completely abolished (Fig. 3B). Addition of nucleotides GTP, GDP, ATP or ADP could not restore the ribosomal interactions (data not shown). These results indicate the need for the full length protein for interaction with the ribosome.

To understand if HflX specifically binds 50S or 30S, co-sedimentation experiments were repeated with purified 30S and 50S components, in presence of different nucleotides. Fig. 3C shows that HflX interacts with the 50S subunit in presence of GTP, GDP, ATP or ADP, but not in the absence of nucleotides (apo). Surprisingly, no interaction was found with 30S (Fig. S2, supplementary material), although the protein binds 16S rRNA too.

### Association with 50S stimulates GTP hydrolysis but not ATP hydrolysis

**Fig. 4:**
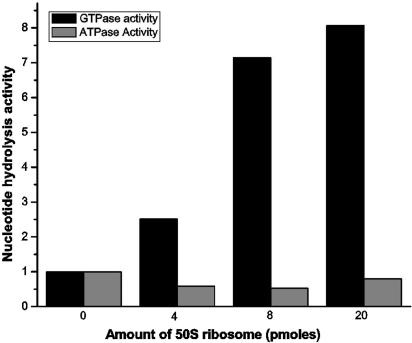
The effect of 50S association on the GTP and ATP hydrolysis activity of HflX. GTP and ATP hydrolysis assays (carried out as in Fig. 2 but in absence of any competing nucleotides) were examined in presence of varying amounts (0–20 pmoles) of 50S. Amount of GTP/ATP hydrolysed, in absence of 50S, was normalized to one to accordingly estimate the fold stimulation.

For GTPases like elongation factor G [20], YjeQ [21] and SRPβ [22], an increase in GTP hydrolysis rate was observed upon ribosome binding. As *E. coli* HflX hydrolyzes both ATP and GTP, and interacts with 50S in the presence of nucleotides (Fig. 3C), the effect of 50S association on the ATP and GTP hydrolysis by HflX was monitored at increasing amounts of ribosome (0–20 pmoles) while protein concentration was held constant. Interestingly, 50S association stimulates GTP hydrolysis rates, while no significant effect was seen on ATP hydrolysis (Fig. 4). At 20 pmoles of 50S, GTP hydrolysis is enhanced by about 8-fold.

## Discussion

*E. coli hfl*X belongs to the *hfl*A operon, which has been implicated in the lysis-lysogeny switch of bacteriophage lambda upon the infection of the bacterium by the phage, mainly from mutational studies. Independent mutational studies have also proposed the involvement of HflX in transposition in *E. coli* [18]. In a recent work (to be published elsewhere), we negate the view that HflX functions in the lambda lysis-lysogeny decision or in the transposition of gene sequences. In this work, we propose a ribosome binding role for HflX.

It was possible to infer a ribosome binding function for HflX, due to ∼40–45% sequence homology between its G domain and that of Obg, DRG1 and Nog1, which bind ribosomal subunits and participate in its biogenesis. While this manuscript was under preparation, association of cpHflX (from *C. pneumonae*) with 50S ribosomal subunits from *E. coli* was reported [17]. In this work, however, we use both HflX and ribosomal subunits from *E. coli*. Our results not only concur with the observations for cpHflX, but also demonstrate that association with 50S requires the presence of a nucleotide. Interestingly, HflX binds and hydrolyses both ATP and GTP, and can associate with 50S in presence of any of the four nucleotides ATP, ADP, GTP or GDP, but not in their absence (i.e., in the apo state) (Fig. 3C). Several GTPases have now been shown to bind ribosomal subunits [7–16] specifically in presence of GTP. The GTP bound state seems to provide an appropriate conformation to promote ribosome binding. Era, however, appears to be the only exception, as it is the apo state that facilitates Era-30S interactions [13]. HflX thus seems to add to the functional diversity of GTPases by associating with 50S in presence of any nucleotide that it binds. In addition, HflX displays a high affinity towards rRNA as indicated by the strong association of the protein with rRNA during purification (data not shown), and a clear association with both 16S and 23S rRNA (Fig. 1). Possibly, an arginine rich region (Fig. S1, Supplementary material) in the N-terminal domain could be important for the RNA interaction.

Sequence analysis reveals that HflX is a three domain protein, of which the N-terminal domain is well conserved among all species and is characterized by the aforesaid glycine rich region (Fig. S1, Supplementary material). We find that all the three domains are required for HflX-ribosome interactions (Fig. 3B). However, its homologues from a few species like *Bacillus subtilis* or *Sulfolobus solfataricus* lack the C terminal domain. Since ΔC-HflX does not interact with 50S (Fig. 3B), it might be that the mode of ribosome binding is different in these homologues.

Based on the presence of sequence motifs G1–G4, HflX is classified as a GTP binding protein, and we find that *E. coli* HflX hydrolyses both ATP and GTP (to be published elsewhere). Unusually, we also find that HflX appears to hydrolyse ATP better than GTP. Interestingly, the competition assays in Fig. 2A, reveal that GTP hydrolysis is inhibited by ATP/AMPPNP, even at a GTP:ATP/AMPPNP ratio of 1:1. A large reduction (∼80%) is noticed when the ratio is 1:25. However, under similar conditions, we could not observe an inhibitory effect on ATP hydrolysis in presence of GTP/GMPPNP (Fig. 2B), although an inhibition may be observed at even higher GTP/GMPPNP concentrations. Based on these findings, it is perhaps reasonable to conclude that both ATP and GTP bind the same site, i.e. the G-domain, and the protein preferentially binds and hydrolyses ATP, over GTP. While the precise roles rendered by ATP and GTP binding/hydrolysis are unclear, cpHflX does not show a similar inhibition of GTP hydrolysis in presence of ATP [17]. This indicates that the function related to ATP binding/hydrolysis may not be common to all HflX homologues.

An increased GTP hydrolysis upon ribosome association is seen for several ribosome binding GTPases. Although HflX hydrolyses both GTP and ATP, 50S association stimulates GTP hydrolysis (∼8-fold), but not ATP hydrolysis (Fig. 4). The present data is insufficient to infer a role for HflX in ribosome biogenesis. However, it is tempting to propose the following model for HflX, which may be different from the roles attributed to other GTPases. 50S binds both the GTP and GDP bound states of HflX, and accelerates GTP hydrolysis. This could lead to a conformational change, either in HflX or the 50S, which may be specifically recognized by the other factor(s). In such a scenario, HflX would act as a helper molecule to promote the binding of other factors that play a direct role in ribosome assembly. Obviously, rigorous experimentation would be required to ascertain the involvement of HflX in the process of ribosome assembly. As ATP hydrolysis is not stimulated by 50S, it may be that the conformations attained by HflX when bound to ATP and GTP are different. Further experiments will be required to confirm the ATPase activity *in vivo*.
