# Supplementary material for: E. coli HflX interacts with 50S ribosomal subunits in presence of nucleotides
Source: Biochem Biophys Res Commun. 2009 Feb 6;379(2):201–5. doi: 10.1016/j.bbrc.2008.12.072 (PMC2686079; doi:10.1016/j.bbrc.2008.12.072)
Supplement: Supplementary material [file mmc1.pdf]

Supplementary Material

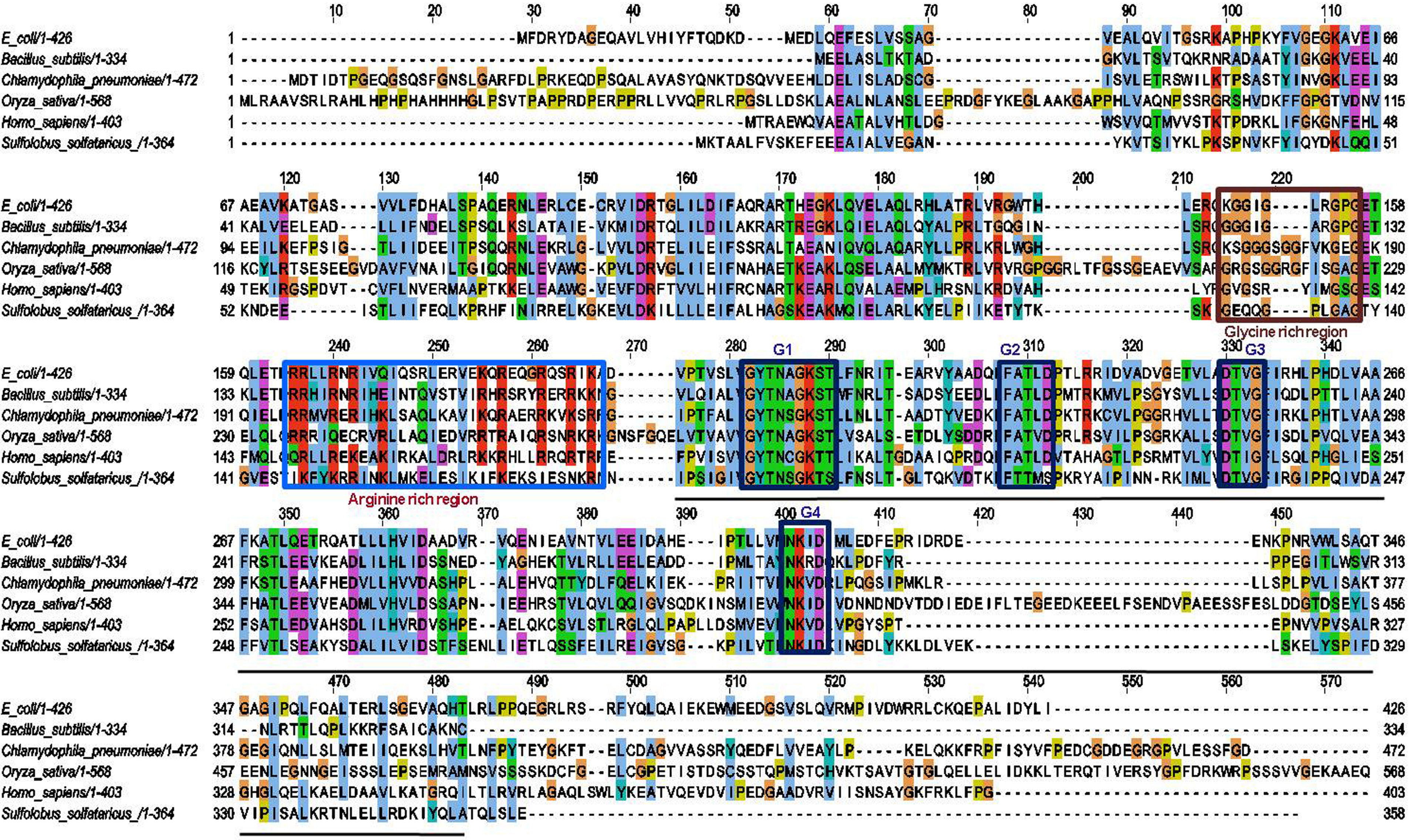

Figure S1: *In-silico* analysis of HflX homologues reveals significant sequence features. Protein sequences belonging to the HflX family, present in all bacteria, eukaryotes, archea but absent in some classes like Mycoplasma, the epsilon subdivision of Proteobacteria, spirochaetes, the archaeon Methanobacterium and fungi, were retrieved from NCBI database and aligned using ClustalX [1]. A Multiple sequence alignment (MSA) of representative sequences, modified manually using Jalview alignment editor [2], is shown in the figure. Domain analysis showed a well defined G domain (region underlined) containing the conserved sequence motifs G1-G4 (boxed), known to be required for GTP binding. Preceding the G domain is a conserved N terminal domain of ~190 amino acids that has no significant sequence similarity to any well characterized domain. It contains a glycine rich region (boxed), which appears to be unique to Obg and HflX families. Sandwiched between the glycine rich region and the G domain, is an arginine rich region (boxed), which presents a positively charged surface that may perhaps serve as a binding site for the rRNA that HflX interacts with. C-terminal domain that follows the G domain (363-426) is not present in some of the orthologs. The numbers refer to the residue numbers in *E. coli* HflX.

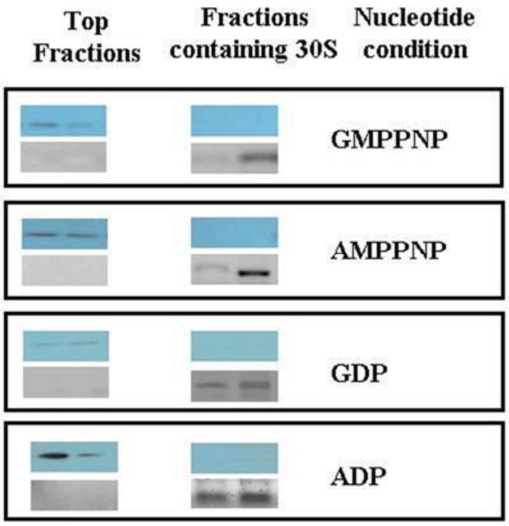

Figure S2: Co-fractionation experiments, as in Fig 3C, were repeated with purified HflX and the 30S subunit in presence of various nucleotides (shown in separate panels), as indicated on the right. Like in Fig 3C, the top fractions devoid of 30S and only the peak fractions containing 30S are shown (top gel, in blue color) based on the presence of 16S rRNA in these fractions (lower gel in grayscale). HflX does not co-fractionate with the 30S, in any of the conditions tested.

Cloning, Expression and Purification of HflX

E. coli hflX gene was amplified and cloned in pET28a vector (Novagen) using primers, given in table S1 (see below), containing BamHI and XhoI restriction sites. For overexpression of recombinant proteins, *E. coli* BL21 cells containing the recombinant plasmid was grown at 37°C and was induced with 0.4mM IPTG (Sigma-Aldrich) at 0.6 $OD_{600.}$ Culture was harvested by centrifugation at 4000 X g at 4°C for 10 minutes, after 8 hours of incubation at 25°C.

Similarly ΔN-HflX lacking 192 amino acids at the N-terminal, ΔC HflX lacking 62 amino acids at the C-terminal and HflX-G (amino acids 193-362) constructs were cloned in pQE2 vector (Qiagen) with NdeI and HindIII restriction sites using appropriate primers (see table S1). DH5α cells carrying the recombinant plasmid were grown at 37°C and induced at 0.6 OD_{600} with 0.2mM IPTG for over-expression. Cultures were harvested by centrifugation at 4000 X g at 4°C for 10 minutes after 12 hours of incubation at 17°C.

Cell pellet was lysed by 5 cycles of freeze-thaw in lysis buffer A {20mM Tris-HCl pH 8, 500mM NaCl, 5% Glycerol, 1mg/ml lysozyme, 3mM β-mercaptoethanol, 1mM PMSF, protease inhibitor cocktail (Sigma-Aldrich)}. This was followed by DNase and RNase treatment. Lysates thus obtained were centrifuged at 35,000 X g at 4°C for 1hour in 50mL oak-ridge tubes (Sorvall SS-34 rotor). Clarified supernatant was loaded on a 5mL Global His-trap affinity column (Amersham) equilibrated with buffer B (20mM Tris-HCl pH 8.0 at 4°C, 500mM NaCl, 5% glycerol 3mM β-meracptoethanol). The column was washed with 100mL of washing buffer B. A linear gradient of imidazole (0-500mM, in buffer B) was used to elute the protein. Eluted fractions were analyzed by SDS PAGE and concentrated with Millipore Amicon ultra centrifugal filter tubes (30KDa cutoff).

Concentrated protein sample was further purified by size exclusion chromatography using superdex200 column (Amersham). The protein eluted as a monomer. Fractions containing the protein were concentrated using Millipore Amicon ultra centrifugal filter tubes (10KDa cutoff), aliquoted and stored at -80°C after snap freezing in liquid nitrogen.

Table S1: Oligos used for cloning the HflX constructs

S.No	Oligo Name	Restriction site§	Sequence (5'-3')
1	HflX1S*	BamHI	GCGGATGGATCCTTGTTTGACCGTTATGA
2	HflX1S*	NdeI	CGCCATATGTTGTTTGACCGTTATGATGC
3	HflX193S	NdeI	CGCCATATGATCAAGCCGACGTTCCTAC
4	HflX 362A*	HindIII	CCCAAGCTTCTAAAGCCGCTTCCGTCAAAGC
5	HflX426A*	HindIII	CGCAAGCTTCTAGATCAGGTAATCGATCAA
6	HflX426A*	XhoI	GCTCTGCCTCGAGCGTTAGATGAGGTAATC′

*Sense and antisense strands are indicated by S and A, respectively.

§ Restriction sites used for cloning (underlined in sequence).

Numbers (in the oligo names) indicate the amino acid in the primary sequence of *E. coli* HflX.

References:

[1]. J.D. Thompson, T.J. Gibson, F. Plewniak, F. Jeanmougin, and D.G. Higgins, The CLUSTAL_X windows interface: flexible strategies for multiple sequence alignment aided by quality analysis tools. Nucl. Acids Res. 25 (1997) 4876-4882.

[2] M. Clamp, J. Cuff, S.M. Searle, and G.J. Barton, The Jalview java alignment editor. Bioinformatics 20 (2004) 426-427.
